# Comparative evaluation of Allplex HPV28 and Anyplex II HPV28 assays for high-risk HPV genotyping in cervical samples

**DOI:** 10.1371/journal.pone.0320978

**Published:** 2025-04-01

**Authors:** Sarah Mafi, Flavie Theuillon, Sylvain Meyer, Jean-Baptiste Woillard, Marine Dupont, Sylvie Rogez, Sophie Alain, Sébastien Hantz

**Affiliations:** 1 Department of Bacteriology, Virology and Hygiene, CHU Limoges, Limoges, France; 2 University of Limoges, INSERM, RESINFIT, U1092, Limoges, France; 3 Department of Pharmacology, Toxicology and Pharmacovigilance, CHU Limoges, Limoges, France; Ruđer Bošković Institute: Institut Ruder Boskovic, CROATIA

## Abstract

**Background/Objectives:**

Human papillomavirus (HPV) genotyping is essential for cervical cancer screening and prevention. The Allplex^TM^ HPV28 real-time PCR kit, using different chemistry and results analysis compared with its predecessor, the Anyplex^TM^ II HPV28 kit, has recently been launched. This study aims to compare the Allplex^TM^ HPV28 and Anyplex^TM^ II HPV28 assays in detecting and genotyping the 13 high-risk (HR)-HPV types.

**Study design:**

Between 2022 and 2023, 459 cervical samples from women undergoing cervical cancer screening were selected. These samples were analysed by liquid-based cytology and tested by both kits concurrently.

**Results:**

Allplex^TM^ HPV28 Ct values correlated well with Anyplex^TM^ II HPV28 signal intensity scores. No significant differences between assays were observed in overall and genotype-specific HR-HPV prevalence determined in all samples and according to cytological results. In addition, no significant differences were identified between assays in the detection of single and multiple HR-HPV infections. Most of the discordant results corresponded to samples showing weak HR-HPV signals and multiple HR-HPV types.

**Conclusions:**

Our results demonstrate that the Allplex^TM^ HPV28 kit can be used for HPV genotyping, with results overall similar to those obtained with the Anyplex^TM^ II HPV28 kit and the addition of Ct values for patient follow-up. The clinical implications of the potentially reduced sensitivity of the Allplex^TM^ HPV28 kit in detecting HPV31 (*p* =  0.07) and HPV39 (*p* =  0.08) warrant further investigation in subsequent studies.

## 1. Introduction

Cervical cancer is the fourth most frequently diagnosed cancer in women worldwide, and the fourth leading cause of cancer death in women [1]. Persistent genital infection with carcinogenic human papillomavirus (HPV) genotypes is a necessary but not sufficient cause of invasive cervical cancer [2]. More than 200 HPV types have been identified, including approximately 40 that preferentially infect the mucosal epithelium [3]. Genital HPV have been subdivided into low-risk (LR-HPV) types, which are principally found in non-malignant lesions, and high-risk (HR-HPV) types, which are associated with the development of cervical intraepithelial neoplasia and invasive cervical cancer. According to the International Agency for Research on Cancer (IARC), 12 carcinogenic types (16, 18, 31, 33, 35, 39, 45, 51, 52, 56, 58, 59) are classified as Group 1, and one probably carcinogenic type (68) is classified as Group 2A [4]. These 13 HPV types are collectively referred to as HR-HPV types in screening assays. HPV16 and 18 are responsible for at least 70% of cervical cancers [5,6]. Moreover, some studies have suggested that co-infections with multiple HPV types may play a role in the development or progression of cervical neoplasia [7–9].

Molecular methods for HPV detection and genotyping, as an adjunct to cytology, therefore play a crucial role in cervical cancer screening, management, and treatment. In addition, they are widely used in epidemiological studies, HPV surveillance programs, and vaccination impact monitoring. Several molecular test kits for primary cervical cancer screening are now available on the market [10], and it is important to validate HPV tests, which guarantee high-quality screening. To this end, guidelines for HPV DNA test requirements have been established by Meijer et al. [11], and an international collaboration, VALGENT (VALidation of HPV GENotyping Tests), was designed to support clinical validation and comparison of HPV assays [12]. To date, three VALGENT study rounds were completed and validation of the Anyplex™ II HPV HR assay (Seegene, Korea) was performed in all three rounds. In our laboratory, the Anyplex™ II HPV28 assay has been used since 2013 for cervical cancer screening (following internal method validation), for assessing the persistence of HPV types, and for monitoring vaccine-targeted HPV types. The primer set A of the Anyplex™ II HPV28 assay targets the 13 carcinogenic or probably carcinogenic HR-HPV types, similar to those detected by the Anyplex™ II HPV HR assay. Recently, a new multiplex real-time PCR kit from the same company, the Allplex^TM^ HPV28 assay, using different chemistry and results analysis, has been launched. The set A of the Allplex™ HPV28 assay is also similar to the Allplex™ HPV HR assay. For the Allplex™ HPV HR assay, updated and clinically validated cut-offs provided and recommended by the manufacturer are applied for each individual HPV genotype based on cycle threshold (Ct) values, whereas positivity for the Allplex^TM^ HPV28 assay is defined by an absolute cut-off of Ct ≤  43. This study aims to compare the Allplex^TM^ HPV28 and Anyplex™ II HPV28 assays in detecting and genotyping the 13 HR-HPV types included in set A of each assay, using a selection of 459 cervical samples.

## 2. Study design

### 2.1. Sample collection

**Fig 1:**
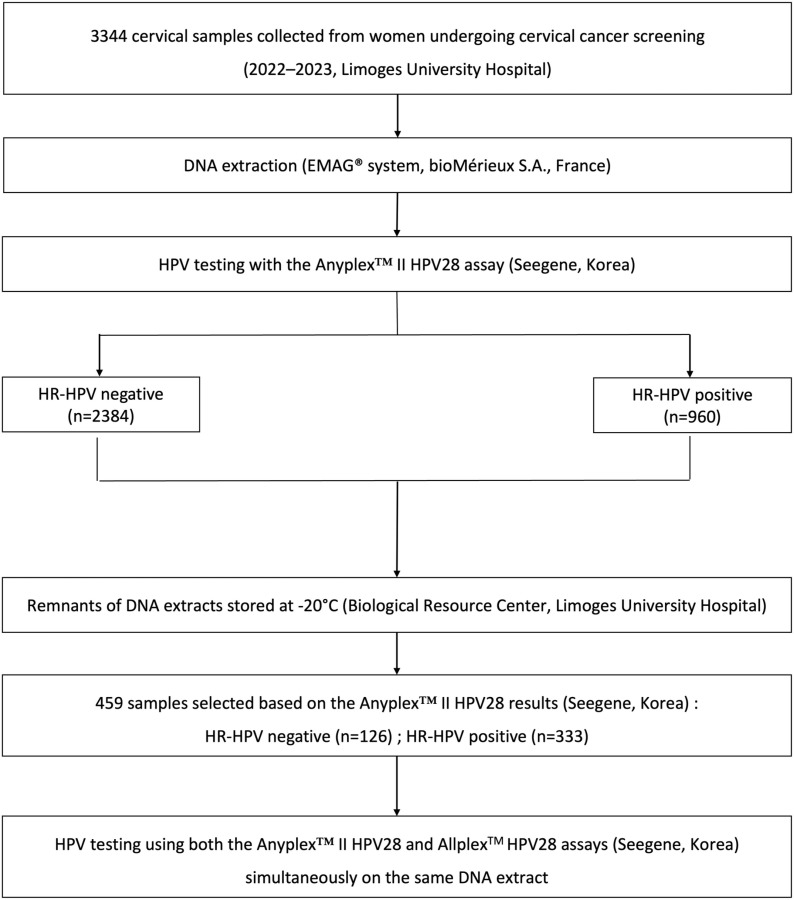
Flow chart of the study design.

The study was performed at the Virology Department of the Limoges University Hospital, France. Between 2022 and 2023, 459 cervical samples, collected in ThinPrep^®^ Pap Test PreservCyt solution (Hologic, USA) from women undergoing cervical cancer screening, were selected (Fig 1). Sample selection was based on the results of the Anyplex^TM^ II HPV28 assay, routinely used in our laboratory for HPV testing. The selection process aimed to obtain a varied panel of screening samples, with at least 20 positive samples for each HR-HPV type. This study was conducted in accordance with the institution’s procedure (USA-P-015 B) for the reuse of health data for research purposes. Patients were informed of the possibility of reuse of their health data and biological samples for research purposes, and could object to this. All data were fully anonymized (no information that could identify individual participants during or after data collection). Data were accessed for research purposes from February 10, 2022, to August 31, 2023.

### 2.2. HPV testing

#### 2.2.1. DNA extraction and storage.

DNA was extracted from 800 µL of each sample using the EMAG^®^ system (bioMérieux S.A., France), which is already implemented in our laboratory for a large number of molecular analyses. Given that this extraction system does not appear in the instructions for use (IFU), rigorous internal method validation was conducted in our laboratory prior to its use. The samples were initially tested with the Anyplex^TM^ II HPV28 kit routinely used in our laboratory. Remnants of DNA extracts were stored at -20°C in the biological collection of the Limoges University Hospital (CRBioLim, ISO 20 387 certified).

#### 2.2.2. HPV PCR assays.

The samples selected based on the results of the Anyplex^TM^ II HPV28 assay were subsequently tested again at the same time (using the same thawed extract) with both the Anyplex^TM^ II HPV28 and Allplex^TM^ HPV28 kits to avoid any storage-related discrepancies. Both multiplex real-time PCR kits are designed to simultaneous amplify, detect and differentiate 28 HPV types in a single reaction. Based on Seegene’s proprietary DPO^TM^ (dual priming oligonucleotide primer) and TOCE^TM^ (tagging oligonucleotide cleavage and extension) technologies, the Anyplex^TM^ II HPV28 assay uses cyclic-CMTA (catcher melting temperature analysis), whereas the Allplex^TM^ HPV28 assay uses a different chemistry (MuDT^TM^ technology, Seegene, combined with DPO™ and TOCE™). Each assay includes two sets of primers. The primer set A detects 12 HR-HPV types (16, 18, 31, 33, 35, 39, 45, 51, 52, 56, 58, 59) classified as Group 1 (carcinogenic to humans), HPV68 classified as Group 2A (probably carcinogenic to humans), and HPV66 classified as Group 2B (possibly carcinogenic to humans). The primer set B detects 14 additional HPV types (6, 11, 26, 40, 42, 43, 44, 53, 54, 61, 69, 70, 73, 82), classified as either Group 2B or Group 3, the latter including types that are not classifiable regarding their carcinogenicity to humans. Briefly, 5 μl of extracted DNA in 15 µL of reaction mixture was amplified with the primer set A. PCR set-up was performed using the ESTREAM^®^ pipetting instrument (bioMérieux S.A., France). DNA amplification was performed using the CFX96^TM^ real-time PCR system (Bio-Rad, USA), according to the on-label use described in the IFU. The human β-globin gene (endogenous internal control, IC) was co-amplified with the *L1* gene of targeted HPV types within the clinical specimens. PCR-generated data were automatically analysed using the Seegene software. For the Anyplex^TM^ II HPV28 assay, signal intensity was categorized as high (+++), intermediate (++), or low (+) for each HPV detected, corresponding to Ct values of <  31, between 31 and 40, or ≥  41, respectively. For the Allplex^TM^ HPV28 assay, individual Ct values were reported, with a positivity threshold defined as Ct values ≤  43, according to the IFU. If the IC was not detected (Ct >  43 or non-amplifiable with the Allplex^TM^ HPV28 assay, or a negative result (-) with the Anyplex^TM^ II HPV28 assay), the test was considered invalid. Detection limit for sensitivity was 50 copies/reaction for both assays (manufacturer data).

### 2.3. Liquid-based cytology

Cervical liquid-based cytology (LBC) was routinely performed following HPV genotyping. LBC slides were stained following the standard laboratory protocol, and cytological diagnoses were performed by experienced pathologists. Results were reported according to the 2001 Bethesda System. Cytological results were retrospectively extracted from medical records to conduct the comparative evaluation of the Allplex^TM^ HPV28 and Anyplex^TM^ II HPV28 assays.

### 2.4. Statistical analysis

Only the 13 HR-HPV types (16, 18, 31, 33, 35, 39, 45, 51, 52, 56, 58, 59, 68) amplified using the primer set A of both assays were considered for analysis. Although HPV66 was also amplified with set A, it was excluded from the analysis as it is currently classified as possibly carcinogenic to humans (Group 2B) with no attributable fraction for cervical cancer [4]. Based on the HR-HPV positivity rates observed for the Allplex^TM^ HPV28 and Anyplex^TM^ II HPV28 assays, a sample size of 459 was determined to be sufficient to detect potential performance differences between each assay, as confirmed using the prop.test() function in R, with a 95% confidence level and an equivalence margin of 10%. There were no invalid results among the 459 samples analysed. The distribution of Allplex^TM^ HPV28 Ct values according to each Anyplex^TM^ II HPV28 signal intensity score was analysed. The Kruskal-Wallis test was used to determine whether there was a statistically significant difference between the medians of the three groups. Overall and genotype-specific HR-HPV prevalence was determined in all samples, and stratified according to cytological results. Agreement between the Allplex^TM^ HPV28 and Anyplex^TM^ II HPV28 assays for overall HR-HPV types, as well as for each HR-HPV type, was assessed by overall percent agreement, positive percent agreement, negative percent agreement, and Cohen’s kappa coefficient calculation (*Κ*). *Κ* values were interpreted as follows: ≤  0.0 indicating no agreement, 0.01–0.20 as none to slight agreement, 0.21–0.40 as fair agreement, 0.41–0.60 as moderate agreement, 0.61–0.80 as substantial agreement, 0.81–0.99 as almost perfect agreement, and 1 as perfect agreement. The McNemar test was used to determine whether detection differences (overall and for each HR-HPV type) between the Allplex^TM^ HPV28 and Anyplex^TM^ II HPV28 assays were statistically significant for paired data. Multiple HR-HPV infection was defined as two or more HR-HPV types detected. The prevalence of overall HR-HPV was analysed in single and multiple HPV infections. The chi-squared test was used to determine whether there was an association between the results obtained with the Allplex^TM^ HPV28 and Anyplex^TM^ II HPV28 assays regarding HR-HPV infection status. Additionally, the McNemar test was used to determine whether detection differences in HR-HPV infection status between the Allplex^TM^ HPV28 and Anyplex^TM^ II HPV28 assays were statistically significant for paired data. Discordant results were analysed according to three variables: sample DNA quality (Allplex^TM^ HPV28 Ct values or Anyplex^TM^ II HPV28 intensity scores for IC), HR-HPV signal intensity (Allplex^TM^ HPV28 Ct values or Anyplex^TM^ II HPV28 intensity scores for HR-HPV), and HR-HPV infection status (single versus multiple HR-HPV infection). The chi-squared test was used to determine whether discordant results between the Allplex™ HPV28 and Anyplex™ II HPV28 assays were associated with HR-HPV infection status. Tables for statistics were generated using Microsoft Excel. Statistics were performed using R (version 4.4.2, R Foundation for Statistical Computing, Vienna, Austria) with R studio (2024.09.1 + 394), Prism 9 software (GraphPad Software, USA), the BiostaTGV website, and MedCalc statistical software (version 22.026, MedCalc Software Ltd., Ostend, Belgium). Statistical differences were considered significant at *p < * 0.05.

## 3. Results

### 3.1. Cytological analysis of cervical samples

**Fig 2:**
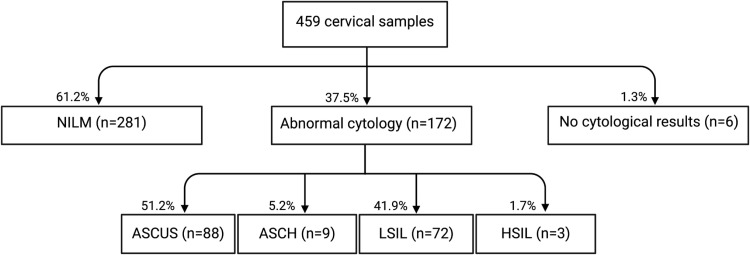
Distribution of cervical cytology results in the study population. NILM (Negative for Intraepithelial Lesion or Malignancy), ASCUS (atypical squamous cells of undetermined significance), ASCH (atypical squamous cells, cannot exclude high-grade squamous intraepithelial neoplasia), LSIL (low-grade squamous intraepithelial lesion), HSIL (high-grade squamous intraepithelial lesion).

LBC results were available for 98.7% of samples (Fig 2). NILM (Negative for Intraepithelial Lesion or Malignancy) results were reported in 61.2% of samples, while abnormal cytology results were observed in 37.5% of samples. Among samples with abnormal cervical cytology, 51.2% were categorized as ASCUS (atypical squamous cells of undetermined significance), 5.2% as ASCH (atypical squamous cells, cannot exclude high-grade squamous intraepithelial neoplasia), 41.9% as LSIL (low-grade squamous intraepithelial lesion), and 1.7% as HSIL (high-grade squamous intraepithelial lesion). No invasive cervical cancer was detected on cytological examination.

### 3.2. Correlation between Allplex^TM^ HPV28 Ct values and Anyplex^TM^ II HPV28 signal intensity scores for HR-HPV

**Fig 3:**
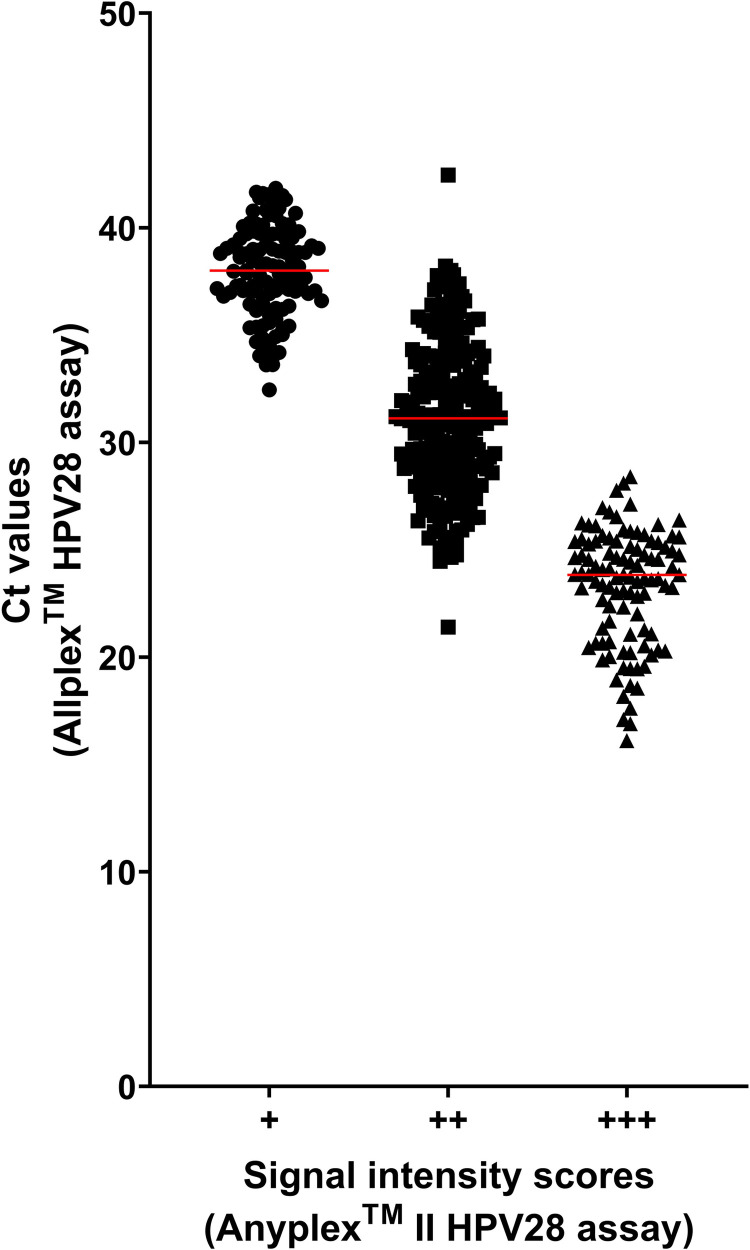
Distribution of Allplex^TM^ HPV28 Ct values according to Anyplex^TM^ II HPV28 signal intensity scores. The correlation between Allplex^TM^ HPV28 Ct values and Anyplex^TM^ II HPV28 signal intensity scores (low [+], circles; intermediate [++], squares; high [+++], triangles) was analysed for all 13 HR-HPV types. Red lines indicate the median Ct value for each signal intensity score.

Allplex^TM^ HPV28 Ct values correlated well with Anyplex^TM^ II HPV28 signal intensity scores, with median Ct values [interquartile range] of 38.0 [36.8-39.6], 31.1 [28.6-33.7], and 23.8 [21.1-25.4] corresponding to + , ++, and +++ signal intensity scores, respectively (Fig 3). There was a statistically significant difference between the medians of each group (*p < * 0.0001, Kruskal-Wallis test).

### 3.3. Overall HR-HPV prevalence

Considering all 13 HR-HPV types, HPV DNA was detected in 72.8% (334/459) and 72.5% (333/459) of samples with the Allplex^TM^ HPV28 and Anyplex^TM^ II HPV28 assays, respectively (Table 1). Overall agreement between assays was 95.0% (436/459). Positive and negative percent agreements were 96.7% (322/333) and 90.5% (114/126), respectively. The kappa value (*K*) was 0.87, indicating an almost perfect agreement between assays. There was no statistically significant difference in overall HR-HPV detection between assays (*p* =  1.00, McNemar’s test for paired data).

**Table 1:** Comparison of the Allplex^TM^ HPV28 and Anyplex^TM^ II HPV28 assays for HR-HPV DNA detection.

		Allplex^TM^ HPV28		
		Positive	Negative	Total
**Anyplex**^**TM**^ **II HPV28**	**Positive**	322	11	333
	**Negative**	12	114	126
	**Total**	334	125	459

### 3.4. Overall HR-HPV prevalence in single and multiple HPV infections

**Fig 4:**
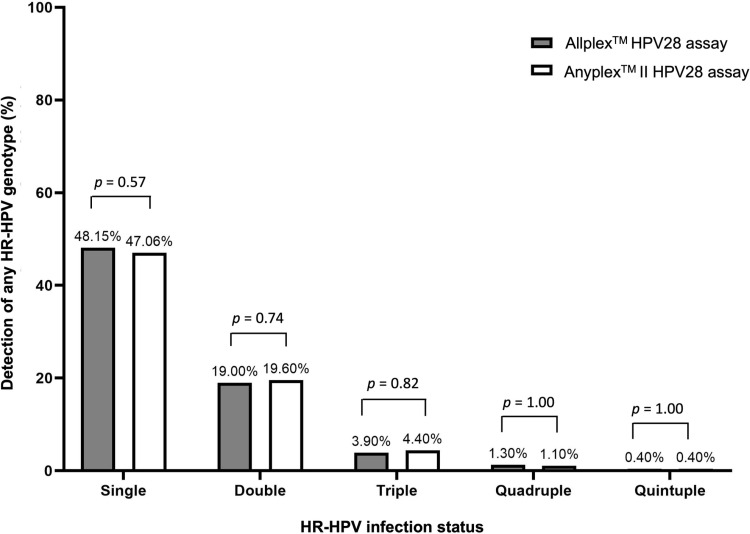
Comparison of the proportion of detection of single and multiple HR-HPV infections (double, triple, quadruple, quintuple) between the Allplex^TM^ HPV28 and Anyplex^TM^ II HPV28 assays.

Based on the results of the Allplex^TM^ HPV28 assay, single and multiple HR-HPV infections were detected in 48.1% (221/459) and 24.6% (113/459) of samples, respectively (Table 2). Based on the results of the Anyplex^TM^ II HPV28 assay, single and multiple HR-HPV infections were detected in 47.1% (216/459) and 25.5% (117/459) of samples, respectively. Chi-square analysis showed a significant association between the Allplex^TM^ HPV28 and Anyplex^TM^ II HPV28 assays for HR-HPV infection status (χ² (4) =  646, 68, *p* <  0.001). Further analysis using the McNemar test for paired data revealed no significant differences in the detection of single and multiple infections (double, triple, quadruple, or quintuples infections) between the Allplex^TM^ HPV28 and Anyplex^TM^ II HPV28 assays (*p* >  0.05) (Fig 4, S1 Table).

**Table 2:** Comparison of the Allplex^TM^ HPV28 and Anyplex^TM^ II HPV28 assays for HR-HPV infection status (single, multiple, and negative cases).

	HR-HPV infection status	Allplex^TM^ HPV28			
		Single	Multiple	Negative	Total
**Anyplex**^**TM**^ **II HPV28**	**Single**	194	11	11	216
	**Multiple**	16	101	0	117
	**Negative**	11	1	114	126
	**Total**	221	113	125	459

### 3.5. Overall HR-HPV prevalence according to cytological results

Considering all 13 HR-HPV types, HPV DNA was detected in 65.5% (184/281) of NILM samples, 83.0% (73/88) of ASCUS samples, 100% (9/9) of ASCH samples, and 100% (3/3) of HSIL samples with both assays (*p > * 0.05, McNemar’s test for paired data) (Table 3). In the LSIL group, HPV DNA was detected in 81.9% (59/72) and 80.6% (58/72) of samples with the Allplex^TM^ HPV28 and Anyplex^TM^ II HPV28 assays, respectively (*p > * 0.05, McNemar’s test for paired data) (Table 3). Of note, the 6 samples for which cytological results were not available were HR-HPV-positive with both assays.

**Table 3:** Comparison of the Allplex^TM^ HPV28 and Anyplex^TM^ II HPV28 assays for the detection of HR-HPV infections in samples with NILM cytology, ASCUS, ASCH, LSIL and HSIL.

Cervical cytology	Population (N = 453^*^)						*p*
	All+ n (%)	Any+ n (%)	All+/Any+ n	All+/Any- n	All-/Any+ n	All-/Any- n	
**NILM (N = 281)**	184 (65.5)	184 (65.5)	174	10	10	87	1.00
**ASCUS** **(N = 88)**	73 (83.0)	73 (83.0)	72	1	1	14	1.00
**ASCH** **(N = 9)**	9 (100)	9 (100)	9	0	0	0	NA
**LSIL** **(N = 72)**	59 (81.9)	58 (80.6)	58	1	0	13	1.00
**HSIL** **(N = 3)**	3 (100)	3 (100)	3	0	0	0	NA

* Cytological results were available for 453/459 samples.

All+, positive with Allplex^TM^ HPV28; Any+, positive with Anyplex^TM^ II HPV28; All+/Any+, positive with both assays; All+/Any-, Allplex^TM^ HPV28 positive and Anyplex^TM^ II HPV28 negative; All-/Any+, Allplex^TM^ HPV28 negative and Anyplex^TM^ II HPV28 positive; All-/Any-, negative with both assays. *p*, McNemar’s test for paired data. NA =  not applicable (if no discordances).

### 3.6. Genotype-specific HR-HPV prevalence according to cytological results

**Fig 5:**
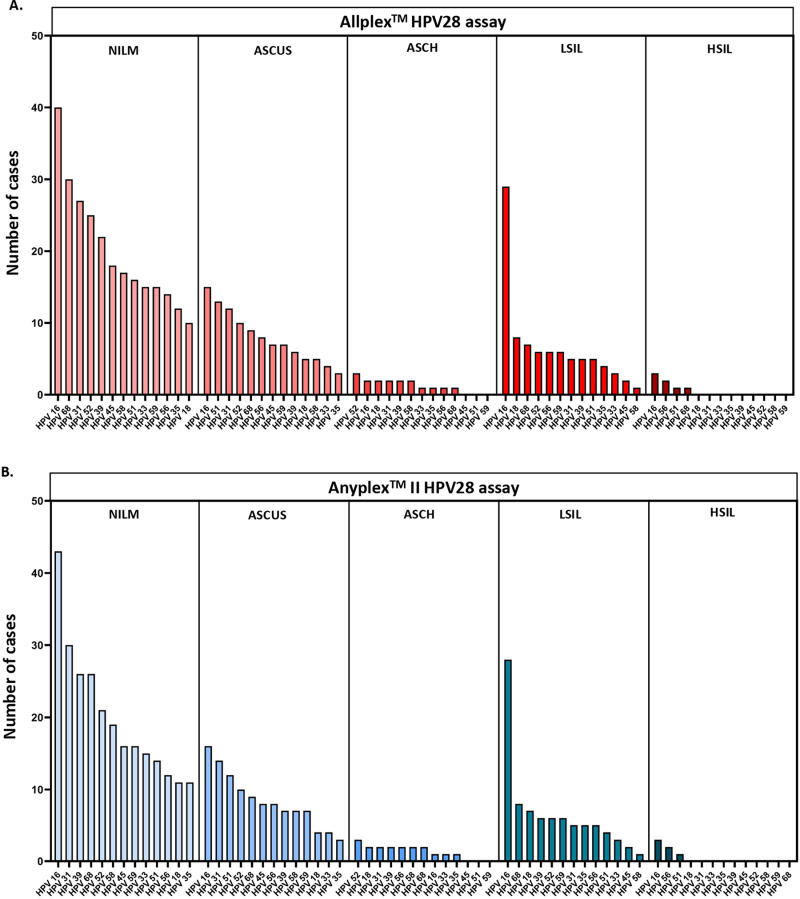
Distribution of HR-HPV types in NILM and abnormal (ASCUS, ASCH, LSIL, HSIL) cytological samples with the Allplex^TM^ HPV28 (A) and Anyplex^TM^ II HPV28 (B) assays. NILM (Negative for Intraepithelial Lesion or Malignancy), ASCUS (atypical squamous cells of undetermined significance), ASCH (atypical squamous cells, cannot exclude high-grade squamous intraepithelial neoplasia), LSIL (low-grade squamous intraepithelial lesion), HSIL (high-grade squamous intraepithelial lesion).

Based on the results of the Allplex^TM^ HPV28 assay, the most prevalent HR-HPV types were: HPV16, HPV68, HPV31, HPV52, and HPV39 in women with NILM cytology; HPV16, HPV51, HPV31, HPV52, and HPV68 in women with ASCUS; HPV52 in women with ASCH; HPV16, HPV18, and HPV68 in women with LSIL; HPV16, and HPV56 in women with HSIL (Fig 5A, S2–S6 Tables).

Based on the results of the Anyplex^TM^ II HPV28 assay, the most prevalent HR-HPV types were: HPV16, HPV31, HPV39, HPV68, and HPV52 in women with NILM cytology; HPV16, HPV31, HPV51, HPV52, and HPV68 in women with ASCUS; HPV52 in women with ASCH; HPV16, HPV68, and HPV18 in women with LSIL; HPV16, and HPV56 in women with HSIL (Fig 5B, S2–S6 Tables).

There was no significant difference between assays in the distribution of each HR-HPV type in NILM and abnormal cytological samples (ASCUS, ASCH, LSIL, and HSIL) (*p > * 0.05, McNemar’s test for paired data) (S2–S6 Tables).

### 3.7. Genotype-specific comparison of the Allplex^TM^ HPV28 and Anyplex^TM^ II HPV28 assays

For each HR-HPV type, overall agreement between assays ranged from 96.7% to 99.6% (Table 4). Positive and negative percent agreements were at least 75.9% and 97.8%, respectively. Genotype-specific agreement was almost perfect, except for HPV58 (substantial). For the 13 HR-HPV types, discordant results were observed in 81 cases (some discordances involving different types were found in identical samples): 39 cases (48.1%) were positive only with Allplex^TM^ HPV28 (Allplex+/Anyplex-), while 42 (51.9%) were positive only with Anyplex^TM^ II HPV28 (Allplex-/Anyplex+). Differences in the detection of each HR-HPV type were not statistically significant (*p* >  0.05, McNemar’s test for paired data). Of note, the Allplex^TM^ HPV28 assay exhibited a trend toward higher detection of HPV51 (4 cases detected only with the Allplex^TM^ HPV28 versus 0 cases detected with the Anyplex^TM^ II HPV28, *p* =  0.13), and HPV52 (5 cases detected only with the Allplex^TM^ HPV28 versus 1 case detected only with the Anyplex^TM^ II HPV28, *p* =  0.22). Conversely, the Anyplex^TM^ II HPV28 assay showed a trend toward higher detection of HPV31 (5 cases detected only with the Anyplex^TM^ II HPV28 versus 0 cases detected with the Allplex^TM^ HPV28, *p* =  0.07) and HPV39 (7 cases detected only with the Anyplex^TM^ II HPV28 versus 1 case detected only with the Allplex^TM^ HPV28, *p* =  0.08). However, none of these differences reached statistical significance (McNemar’s test for paired data).

**Table 4:** Genotype-specific comparison of the Allplex^TM^ HPV28 and Anyplex^TM^ II HPV28 assays for HR-HPV DNA detection.

	All+ n (%)	Any+ n (%)	Genotype-sample combinations (N = 459)				Overall agreement (%)	PPA (%)	NPA (%)	*K*	^*^Int.	*p*
			All+/Any+ n	All+/Any- n	All-/Any+ n	All-/Any- n						
**HPV 16**	90 (19.6)	92 (20.0)	86	4	6	363	97.8	93.5	98.9	0.93	Almost perfect	0.75
**HPV 18**	25 (5.4)	24 (5.2)	21	4	3	431	98.5	87.5	99.1	0.85	Almost perfect	1.00
**HPV 31**	47 (10.2)	52 (11.3)	47	0	5	407	98.9	90.4	100.0	0.94	Almost perfect	0.07
**HPV 33**	23 (5.0)	23 (5.0)	21	2	2	434	99.1	91.3	99.5	0.91	Almost perfect	1.00
**HPV 35**	21 (4.6)	21 (4.6)	20	1	1	437	99.6	95.2	99.8	0.95	Almost perfect	1.00
**HPV 39**	36 (7.8)	42 (9.2)	35	1	7	416	98.3	83.3	99.8	0.89	Almost perfect	0.08
**HPV 45**	27 (5.9)	26 (5.7)	25	2	1	431	99.3	96.2	99.5	0.94	Almost perfect	1.00
**HPV 51**	36 (7.8)	32 (7.0)	32	4	0	423	99.1	100.0	99.1	0.94	Almost perfect	0.13
**HPV 52**	44 (9.6)	40 (8.7)	39	5	1	414	98.7	97.5	98.8	0.92	Almost perfect	0.22
**HPV 56**	32 (7.0)	30 (6.5)	29	3	1	426	99.1	96.7	99.3	0.93	Almost perfect	0.62
**HPV 58**	25 (5.4)	29 (6.3)	22	3	7	427	97.8	75.9	99.3	0.80	Substantial	0.34
**HPV 59**	28 (6.1)	29 (6.3)	27	1	2	429	99.3	93.1	99.8	0.94	Almost perfect	1.00
**HPV 68**	49 (10.7)	46 (10.0)	40	9	6	404	96.7	87.0	97.8	0.82	Almost perfect	0.61

All+/Any+, positive with both assays; All+/Any-, Allplex^TM^ HPV28 positive and Anyplex^TM^ II HPV28 negative; All-/Any+, Allplex^TM^ HPV28 negative and Anyplex^TM^ II HPV28 positive; All-/Any-, negative with both assays; NPA, negative predictive values; PPA, positive predictive values; *K*, Cohen’s kappa coefficients;

* Int., Interpretation of Cohen’s kappa coefficients (*K*); *p*, McNemar’s test.

### 3.8. Analysis of discordant results according to sample DNA quality (Allplex^TM^ HPV28 Ct values or Anyplex^TM^ II HPV28 intensity scores for IC)

The mean IC Ct values were almost identical between Allplex+/Anyplex- samples and non-discordant samples (25.27 versus 25.20, respectively). Similarly, the IC signal intensities were predominantly categorized as ++ or +++ in both Allplex-/Anyplex+ samples and non-discordant samples, with no significant differences between the groups. These findings support that sample DNA quality was comparable between discordant and non-discordant samples.

### 3.9. Analysis of discordant results according to HR-HPV signal intensity (Allplex^TM^ HPV28 Ct values or Anyplex^TM^ II HPV28 intensity scores for HR-HPV)

**Fig 6:**
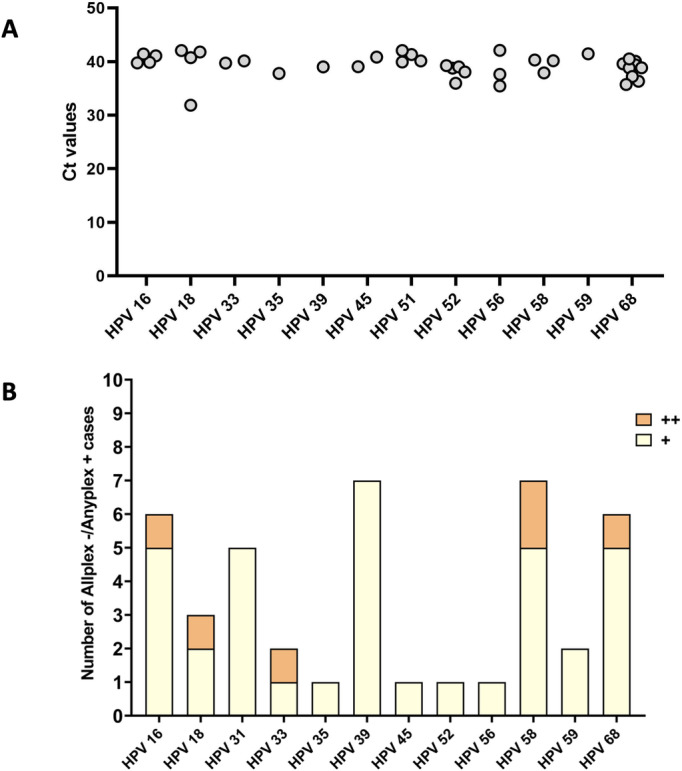
Distribution of Allplex+/Anyplex- results according to Ct values (A). Distribution of Allplex-/Anyplex+ results according to signal intensity scores (B).

Among Allplex+/Anyplex- cases, 97.4% (38/39) had Ct values >  35 (Fig 6A). Among Allplex-/Anyplex+ cases, 85.7% (36/42) were detected at the lowest signal intensity (+), and 14.3% (6/42) at the intermediate signal intensity (++). No Allplex-/Anyplex + cases were detected at high signal intensity (Fig 6B).

### 3.10. Analysis of discordant results according to HR-HPV infection status

**Fig 7:**
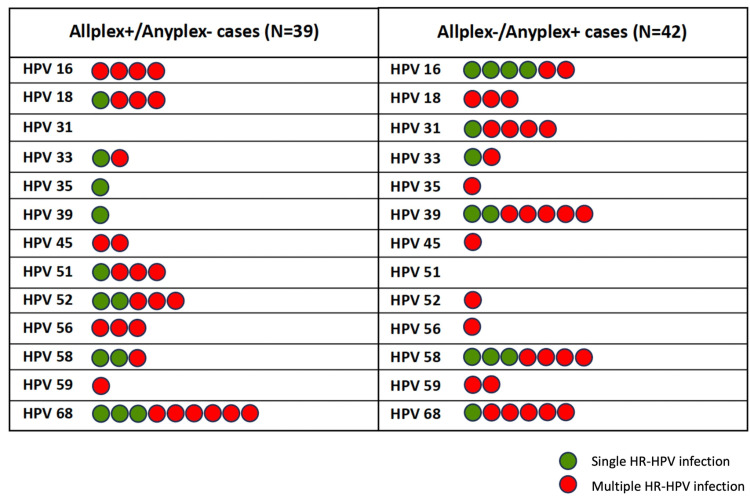
Distribution of Allplex+/Anyplex- and Allplex-/Anyplex+ results per genotype in single and multiple HR-HPV infections.

Although multiple HR-HPV infections were more frequently observed among discordant cases (69.2%, 27/39 in the Allplex+/Anyplex- group and 71.4%, 30/42 in the Allplex-/Anyplex+ group) (Table 5, Fig 7), chi-squared analysis revealed no statistically significant association between discordant results and HR-HPV infection status (χ² (1) =  0.047, *p* =  0.83) (Table 5).

**Table 5:** Comparison of the discordant results between the Allplex^TM^ HPV28 and Anyplex^TM^ II HPV28 assays for the detection of single and multiple HR-HPV infections.

HR-HPV infection status	All+/Any-	All-/Any+	Total
**Single**	12	12	24
**Multiple**	27	30	57
**Total**	39	42	81

All+/Any-, Allplex^TM^ HPV28 positive and Anyplex^TM^ II HPV28 negative; All-/Any+, Allplex^TM^ HPV28 negative and Anyplex^TM^ II HPV28 positive.

## 4. Discussion

HPV genotyping is used in a variety of settings, including cervical cancer screening, clinical management of HPV infections, epidemiological and research studies. Currently, there are more than 250 commercially available HPV assays [10], with different performance characteristics. It is therefore essential to evaluate each HPV test according to its intended use. In particular, clinical validation of HPV assays is required for use in primary cervical cancer screening. Some new HPV assays are designed to simultaneously detect multiple HPV types, and assess their relative abundance. These new assays may be useful to assess the status of infection over time (single versus multiple HPV infections), the correlation between HPV DNA levels and the persistence of specific HPV types, and the progression of cervical dysplasia according to specific HPV types and HPV DNA levels. In the present study, a new multiplex real-time PCR kit, the Allplex^TM^ HPV28 assay, was compared with the Anyplex^TM^ II HPV28 assay for the detection and genotyping of the 13 HR-HPV types.

The association between Ct values obtained with the Allplex^TM^ HPV28 kit and signal intensity scores obtained with the Anyplex^TM^ II HPV28 kit was first investigated and showed a good correlation. The prognostic significance of HR-HPV viral load in cervical cancer remains controversial. Previous studies have reported an association between increased HR-HPV viral load and the severity and progression of cervical lesions [13,14], while others have not found such a correlation [15,16]. Although there is no clear evidence of the value of HPV quantification in cervical cancer screening, it may be relevant to monitor HPV quantification as a potential prognostic marker of residual lesions after treatment [17,18]. In this context, the Allplex^TM^ HPV28 assay, which provides signal strength information through Ct values, may be useful.

The Allplex^TM^ HPV28 and Anyplex^TM^ II HPV28 assays were then compared for HPV DNA detection. We reported an almost perfect agreement between assays for the overall detection of the 13 HR-HPV types (95.0% concordance, *K* = 0.87). Our results are in line with a previous study conducted by Bell M. et al., demonstrating a high concordance between the Allplex^TM^ HPV28 and Anyplex^TM^ II HPV28 assays in mocked self-samples (100% concordance, *K* = 1.00, analysis performed on the 14 HPV types included in set A) [19]. In another study conducted by Oštrbenk Valenčak A. et al., a high overall agreement was reported between the Allplex™ HPV HR and the Anyplex™ II HPV HR assays (96.3% concordance, *K* = 0.88). This agreement was based on clinically validated Ct cut-offs for each individual HPV type, as recommended by the manufacturer for the Allplex™ HPV HR assay: Ct ≤  40 for HPV16 and HPV18, Ct ≤  37 for HPV31, HPV33, HPV45, HPV52 and HPV58, and Ct ≤  35 for HPV35, HPV39, HPV51, HPV56, HPV59, HPV66 and HPV68 (instead of an absolute cut-off of Ct ≤  43 used with the Allplex^TM^ HPV28 assay for all 14 targeted HPV types) [20]. If genotype-specific cut-offs, as implemented for the Allplex™ HPV HR assay, had been applied in our study, the positivity rate for certain genotypes with low viral loads would have been reduced. Indeed, several samples in our study exceeded these cut-offs (Ct >  35). This highlights the importance of carefully evaluating the cut-offs used for cervical cancer screening and HPV infection monitoring, as strict cut-offs may potentially miss low-level infections, even though such infections are not necessarily clinically significant [14]. As no clinical cut-offs were applied for either assay in our study, the comparative analysis between the Allplex™ HPV28 and Anyplex™ II HPV28 assays was not influenced by differences in cut-offs. We found an almost perfect agreement between the Allplex^TM^ HPV28 and Anyplex^TM^ II HPV28 assays for each of the 13 HR-HPV types ( > 96.0% concordance, K = 0.82-0.95), except for HPV58 (97.8% concordance, K = 0.80, substantial agreement). Analysis of discordant results showed no significant difference in the detection of overall or individual HR-HPV type. In contrast, Oštrbenk Valenčak A. et al. reported a statistically significant difference in overall genotype detection (*p* <  0.0001) and for three specific genotypes: HPV31, HPV51, and HPV68 - in favor of the Anyplex™ II HPV HR assay, with p-values of 0.002, 0.02, and 0.008, respectively (using the clinically validated Ct cut-offs) [20]. In our study, we observed a trend toward higher detection of HPV51 and HPV52 with the Allplex^TM^ HPV28 assay compared to the Anyplex^TM^ II HPV28 assay (using the absolute cut-off of Ct ≤  43 for all targeted HPV genotypes), although this difference did not reach statistical significance. Conversely, the Anyplex^TM^ II HPV28 assay showed a trend toward higher detection of HPV31 and HPV39 compared to the Allplex^TM^ HPV28 assay (using the same absolute cut-off), but this also did not reach statistical significance. The internal control signal intensity was comparable between discordant and non-discordant samples, supporting that the observed discordances were not attributable to poor DNA sample quality. Most of our discordant results corresponded to samples with weak HR-HPV signals, suggesting differences in sensitivity between assays according to HR-HPV types. Although both kits target the same gene (HPV *L1* gene), they do not use the same PCR reagents (possible variations in the primer binding sequences and/or probes). However, the location of the primers/probes within HPV genomes is Seegene’s proprietary information, so it is impossible to confirm this hypothesis. Notably, Allplex^TM^ HPV28 primers may target HPV51 and HPV52 more effectively, whereas Anyplex^TM^ II HPV28 primers may be more effective in detecting HPV31 and HPV39. Moreover, a higher rate of discordant results was observed in multiple than in single HPV infections, although no statistically significant association between HR-HPV infection status and discordant results was found. As previously reported with consensus PCR assays, the co-amplification of β-globin with HPV DNA or the presence of multiple HPV types can result in competition for assay reagents and hamper assay performance [21,22]. Nevertheless, the DPO systems for multiplex detection may generate less competition [23].

The role of multiple HPV infections in the progression of cervical disease is still controversial. In some studies, multiple HPV infections were not associated with an increased risk of cervical cancer in women with cytological abnormalities [24–26]. Other studies have suggested a significantly increased risk of cervical carcinogenesis among women with multiple HPV infections compared with women with single infections [7–9]. In this context, the identification of multiple infections by HPV testing remains an important issue. In our study, multiple HPV types were detected in approximately one-third of HR-HPV-infected samples, consistent with previous studies reporting multiple HPV infections in 10% to 50% of HPV-positive women [27–29]. The proportion of multiple infections was not significantly different between the Allplex^TM^ HPV28 and Anyplex^TM^ II HPV28 assays, although a slightly higher number of multiple infections was detected with the Anyplex^TM^ II HPV28 assay. This observation aligns with recent data reporting a slightly higher detection rate of multiple HPV infections using the Anyplex^TM^ II HPV28 assay compared with the Allplex^TM^ HPV28 assay [19].

The prevalence of HR-HPV types was also analysed according to cytological results. Detection rates of HR-HPV (overall or per genotype) was not significantly different between the Allplex^TM^ HPV28 and Anyplex^TM^ II HPV28 assays in NILM or abnormal cytological samples (ASCUS, ASCH, LSIL, HSIL). As expected, a trend towards higher HR-HPV positivity rates was observed as cytological grade increased (from 66% in NILM samples to 81%-100% in ASCUS/ASCH/LSIL/HSIL samples). Considering genotype-specific HR-HPV prevalence, HPV16 was the predominant type in cervical samples with NILM, ASCUS, LSIL and HSIL, in line with previously published data [30]. A summary report on HPV and related diseases in France classified the ten most frequent carcinogenic HPV types among women with NILM, LSIL, HSIL and cervical cancer [31]. According to this report, HPV16, 51, 56, 31, 52, and 39 were the six most frequent genotypes in women with NILM [31]. In the present study, HPV16, 52, 31 and 39 were among the most frequent genotypes in women with NILM (in a different order of frequency between the Allplex^TM^ HPV28 and Anyplex^TM^ II HPV28 assays, but with no significant difference). Our results are therefore broadly consistent with these French data, except for HPV51 and HPV56. In women with LSIL, HPV16, 18, and 68 were the most frequent with the Allplex^TM^ HPV28 and Anyplex^TM^ II HPV28 assays (in a different order of frequency between the Allplex^TM^ HPV28 and Anyplex^TM^ II HPV28 assays, but with no significant difference). HPV18 was the second (Allplex^TM^ HPV28 assay) or third (Anyplex^TM^ II HPV28 assay) most frequent carcinogenic HPV type according to our results, whereas it ranked fourth among the ten most frequent carcinogenic types (after HPV16, 51 and 52) according to French epidemiology [31]. As in women with NILM, a lower prevalence of HPV51 (compared with other carcinogenic HPV) was therefore observed in our study in women with LSIL. In women with HSIL, the most common carcinogenic types were HPV16 and 56 with both assays. According to French data, HPV16, 31, 33, 52, 51 were the most frequent in the HSIL group, and HPV56 was not found among the ten most frequent HPV carcinogenic types [31]. Overall, the differences observed could be explained by geographical disparities in the distribution of HPV types in France. In addition, our study, performed on a smaller sample size (N = 459) compared to the dataset used in the summary report on HPV and related diseases in France [31], is therefore less representative of the broader French population.

The main strength of our study is the number of HR-HPV-positive samples (Allplex^TM^ HPV28 assay, n = 334; Anyplex^TM^ II HPV28 assay, n = 333). Samples were tested simultaneously by both assays, using the same DNA extract stored at -20°C. The differences observed between assays are therefore not related to DNA extraction or storage.

However, this study has some limitations. Firstly, the selection of HR-HPV-positive samples may have introduced a bias and explains the relatively low number of HR-HPV-negative samples. The prevalence of HR-HPV infections (HR-HPV DNA detected in over 70% of cervical samples) is therefore overestimated. Thus, the ranking of genotypes obtained in this study, rather than the prevalence, was compared with data reported in the French population, stratified by cytological subgroup. Nevertheless, the high prevalence of HR-HPV infections and the presence of multiple HR-HPV infections in our population enabled us to increase the statistical power of our analysis for each HR-HPV type (at least 20 positive samples per HR-HPV type). Secondly, the study population was composed of more than 50% ASCUS among samples with abnormal cytology. This high proportion of ASCUS is consistent with the current French cervical cancer screening strategy but does not reflect the general population. Many samples were sent to the laboratory for HR-HPV testing as a triage strategy (women aged 25-29 years with ASCUS diagnoses). Furthermore, the study population included only 1.7% of HSIL among abnormal cervical samples, limiting the statistical power of the subgroup analysis. Additionally, the comparison between the Allplex^TM^ HPV28 and Anyplex^TM^ II HPV28 assays for HR-HPV genotyping could not be assessed in women with cervical cancer (absence of cervical cancer smears). Thirdly, 37.5% of the study population consisted of selected samples with abnormal cytology results. This relatively high frequency of cytological abnormalities, compared to unselected screening samples, may contribute to increase the positive agreement between assays. As suggested by Rebolj M. et al., women with cytological abnormalities may exhibit, on average, higher viral loads than the minimum detectable threshold required for a positive result [32]. In contrast, samples with relatively low viral loads might be more susceptible to the established cut-off on any assay before these assays yield a positive result [32]. Therefore, viral loads in unselected screening samples are likely to be more heterogeneous, encompassing a range from recent transient infections to high-level persistent infections, allowing samples with lower viral loads to have a more significant influence on the frequency of disagreement between the assays [32]. Fourthly, we did not conduct reproducibility studies on the Allplex^TM^ HPV28 assay, which constitutes a limitation in the evaluation of this new assay.

In conclusion, our results demonstrate that the Allplex^TM^ HPV28 assay can be used for HR-HPV detection and genotyping, with results overall similar to those obtained with the well-known Anyplex^TM^ II HPV28 kit and the addition of Ct values for patient follow-up. This finding aligns with the study performed by Oštrbenk Valenčak A. et al., which concluded that the Allplex™ HPV HR assay meets all validation criteria outlined in the international guidelines for HPV testing in primary cervical cancer screening for women aged 30 years and older [20]. Therefore, the use of this new assay may be of great value for cervical cancer screening and HPV infections monitoring. In our study, most discordant results between the Allplex^TM^ HPV28 and Anyplex^TM^ II HPV28 assays corresponded to samples exhibiting weak HR-HPV signals and multiple HR-HPV types. In these samples, the Allplex^TM^ HPV28 assay appears to show a trend toward higher sensitivity in detecting HPV51 and HPV52, whereas HPV31 and HPV39 were more frequently detected with the Anyplex^TM^ II HPV28 assay, although these differences did not reach statistical significance. HPV31 and HPV39 are the third (prevalence of 3.8%) and tenth (prevalence of 0.8%) most frequent carcinogenic genotypes among women with cervical cancer in France, respectively [31]. The clinical implications of the potentially reduced sensitivity of the Allplex^TM^ HPV28 assay in detecting HPV31 (*p* =  0.07) and HPV39 (*p* =  0.08) warrant further investigation in subsequent studies.

## Supporting information

S1 Table Comparison of the Allplex^TM^ HPV28 and Anyplex^TM^ II HPV28 assays for the detection of double, triple, quadruple and quintuple HR-HPV infections. All+, positive with Allplex^TM^ HPV28; Any+, positive with Anyplex^TM^ II HPV28; All+/Any+, positive with both assays; All+/Any-, Allplex^TM^ HPV28 positive and Anyplex^TM^ II HPV28 negative; All-/Any+, Allplex^TM^ HPV28 negative and Anyplex^TM^ II HPV28 positive; All-/Any-, negative with both assays. *p*, McNemar’s test for paired data.
(DOCX)

S2 Table Comparison of the Allplex^TM^ HPV28 and Anyplex^TM^ II HPV28 assays for the specific detection of HR-HPV types in NILM cytological samples. All+, positive with Allplex^TM^ HPV28; Any+, positive with Anyplex^TM^ II HPV28; All+/Any+, positive with both assays; All+/Any-, Allplex^TM^ HPV28 positive and Anyplex^TM^ II HPV28 negative; All-/Any+, Allplex^TM^ HPV28 negative and Anyplex^TM^ II HPV28 positive; All-/Any-, negative with both assays. *p*, McNemar’s test for paired data.
(DOCX)

S3 Table Comparison of the Allplex^TM^ HPV28 and Anyplex^TM^ II HPV28 assays for the specific detection of HR-HPV types in ASCUS samples. All+, positive with Allplex^TM^ HPV28; Any+, positive with Anyplex^TM^ II HPV28; All+/Any+, positive with both assays; All+/Any-, Allplex^TM^ HPV28 positive and Anyplex^TM^ II HPV28 negative; All-/Any+, Allplex^TM^ HPV28 negative and Anyplex^TM^ II HPV28 positive; All-/Any-, negative with both assays. *p*, McNemar’s test for paired data. NA =  not applicable (if no discordances).
(DOCX)

S4 Table Comparison of the Allplex^TM^ HPV28 and Anyplex^TM^ II HPV28 assays for the specific detection of HR-HPV types in ASCH samples. All+, positive with Allplex^TM^ HPV28; Any+, positive with Anyplex^TM^ II HPV28; All+/Any+, positive with both assays; All+/Any-, Allplex^TM^ HPV28 positive and Anyplex^TM^ II HPV28 negative; All-/Any+, Allplex^TM^ HPV28 negative and Anyplex^TM^ II HPV28 positive; All-/Any-, negative with both assays. *p*, McNemar’s test for paired data. NA =  not applicable (if no discordances).
(DOCX)

S5 Table Comparison of the Allplex^TM^ HPV28 and Anyplex^TM^ II HPV28 assays for the specific detection of HR-HPV types in LSIL samples. All+, positive with Allplex^TM^ HPV28; Any+, positive with Anyplex^TM^ II HPV28; All+/Any+, positive with both assays; All+/Any-, Allplex^TM^ HPV28 positive and Anyplex^TM^ II HPV28 negative; All-/Any+, Allplex^TM^ HPV28 negative and Anyplex^TM^ II HPV28 positive; All-/Any-, negative with both assays. *p*, McNemar’s test for paired data. NA =  not applicable (if no discordances).
(DOCX)

S6 Table Comparison of the Allplex^TM^ HPV28 and Anyplex^TM^ II HPV28 assays for the specific detection of HR-HPV types in HSIL samples. All+, positive with Allplex^TM^ HPV28; Any+, positive with Anyplex^TM^ II HPV28; All+/Any+, positive with both assays; All+/Any-, Allplex^TM^ HPV28 positive and Anyplex^TM^ II HPV28 negative; All-/Any+, Allplex^TM^ HPV28 negative and Anyplex^TM^ II HPV28 positive; All-/Any-, negative with both assays. *p*, McNemar’s test for paired data. NA =  not applicable (if no discordances).
(DOCX)
