# Supplementary material for: Comparative evaluation of Allplex HPV28 and Anyplex II HPV28 assays for high-risk HPV genotyping in cervical samples
Source: PLoS One. 2025 Apr 1;20(4):e0320978. doi: 10.1371/journal.pone.0320978 (PMC11960881; doi:10.1371/journal.pone.0320978)
Supplement: S1 Table — All+, positive with AllplexTM HPV28; Any+, positive with AnyplexTM II HPV28; All+/Any+, positive with both assays; All+/Any-, AllplexTM HPV28 positive and AnyplexTM II HPV28 negative; All-/Any+, AllplexTM HPV28 negative and AnyplexTM II HPV28 positive; All-/Any-, negative with both assays. p, McNemar’s test for paired data. (DOCX) [file pone.0320978.s001.docx]

**S1 Table. Comparison of the Allplex^TM^ HPV28 and Anyplex^TM^ II HPV28 assays for the detection of double, triple, quadruple and quintuple HR-HPV infections.**

| **HR-HPV infection status** | **Population (N=459)** | | | | | |  |
| --- | --- | --- | --- | --- | --- | --- | --- |
|  | **All+**  **n (%)** | **Any+**  **n (%)** | **All+/Any+**  **n** | **All+/Any-**  **n** | **All-/Any+**  **n** | **All-/Any-**  **n** | ***p*** |
| **Double** | 87 (19.0) | 90 (19.6) | 71 | 16 | 19 | 353 | 0.74 |
| **Triple** | 18 (3.9) | 20 (4.4) | 9 | 9 | 11 | 430 | 0.82 |
| **Quadruple** | 6 (1.3) | 5 (1.1) | 1 | 5 | 4 | 449 | 1.00 |
| **Quintuple** | 2 (0.4) | 2 (0.4) | 0 | 2 | 2 | 455 | 1.00 |

All+, positive with Allplex^TM^ HPV28; Any+, positive with Anyplex^TM^ II HPV28; All+/Any+, positive with both assays; All+/Any-, Allplex^TM^ HPV28 positive and Anyplex^TM^ II HPV28 negative; All-/Any+, Allplex^TM^ HPV28 negative and Anyplex^TM^ II HPV28 positive; All-/Any-, negative with both assays. *p*, McNemar’s test for paired data.
