# Supplementary material for: Comparative evaluation of Allplex HPV28 and Anyplex II HPV28 assays for high-risk HPV genotyping in cervical samples
Source: PLoS One. 2025 Apr 1;20(4):e0320978. doi: 10.1371/journal.pone.0320978 (PMC11960881; doi:10.1371/journal.pone.0320978)
Supplement: S2 Table — All+, positive with AllplexTM HPV28; Any+, positive with AnyplexTM II HPV28; All+/Any+, positive with both assays; All+/Any-, AllplexTM HPV28 positive and AnyplexTM II HPV28 negative; All-/Any+, AllplexTM HPV28 negative and AnyplexTM II HPV28 positive; All-/Any-, negative with both assays. p, McNemar’s test for paired data. (DOCX) [file pone.0320978.s002.docx]

**S2 Table. Comparison of the Allplex^TM^ HPV28 and Anyplex^TM^ II HPV28 assays for the specific detection of HR-HPV types in NILM cytological samples.**

| **HPV genotypes** | **Population (N=281)** | | | | | |  |
| --- | --- | --- | --- | --- | --- | --- | --- |
|  | **All+**  **n (%)** | **Any+**  **n (%)** | **All+/Any+**  **n** | **All+/Any-**  **n** | **All-/Any+**  **n** | **All-/Any-**  **n** | ***p*** |
| **HPV 16** | 40 (14.2) | 43 (15.3) | 39 | 1 | 4 | 237 | 0.37 |
| **HPV 18** | 10 (3.6) | 11 (3.9) | 9 | 1 | 2 | 269 | 1.00 |
| **HPV 31** | 27 (9.6) | 30 (10.7) | 27 | 0 | 3 | 251 | 0.25 |
| **HPV 33** | 15 (5.3) | 15 (5.3) | 13 | 2 | 2 | 264 | 1.00 |
| **HPV 35** | 12 (4.3) | 11 (3.9) | 11 | 1 | 0 | 269 | 1.00 |
| **HPV 39** | 22 (7.8) | 26 (9.3) | 21 | 1 | 5 | 254 | 0.22 |
| **HPV 45** | 18 (6.4) | 16 (5.7) | 16 | 2 | 0 | 263 | 0.48 |
| **HPV 51** | 16 (5.7) | 14 (5.0) | 14 | 2 | 0 | 265 | 0.48 |
| **HPV 52** | 25 (8.9) | 21 (7.5) | 20 | 5 | 1 | 255 | 0.22 |
| **HPV 56** | 14 (5.0) | 12 (4.3) | 12 | 2 | 0 | 267 | 0.48 |
| **HPV 58** | 17 (6.0) | 19 (6.8) | 15 | 2 | 4 | 260 | 0.68 |
| **HPV 59** | 15 (5.3) | 16 (5.7) | 14 | 1 | 2 | 264 | 1.00 |
| **HPV 68** | 30 (10.7) | 26 (9.3) | 24 | 6 | 2 | 249 | 0.29 |

All+, positive with Allplex^TM^ HPV28; Any+, positive with Anyplex^TM^ II HPV28; All+/Any+, positive with both assays; All+/Any-, Allplex^TM^ HPV28 positive and Anyplex^TM^ II HPV28 negative; All-/Any+, Allplex^TM^ HPV28 negative and Anyplex^TM^ II HPV28 positive; All-/Any-, negative with both assays. *p*, McNemar’s test for paired data.
