# Supplementary material for: Comparative evaluation of Allplex HPV28 and Anyplex II HPV28 assays for high-risk HPV genotyping in cervical samples
Source: PLoS One. 2025 Apr 1;20(4):e0320978. doi: 10.1371/journal.pone.0320978 (PMC11960881; doi:10.1371/journal.pone.0320978)
Supplement: S3 Table — All+, positive with AllplexTM HPV28; Any+, positive with AnyplexTM II HPV28; All+/Any+, positive with both assays; All+/Any-, AllplexTM HPV28 positive and AnyplexTM II HPV28 negative; All-/Any+, AllplexTM HPV28 negative and AnyplexTM II HPV28 positive; All-/Any-, negative with both assays. p, McNemar’s test for paired data. NA = not applicable (if no discordances). (DOCX) [file pone.0320978.s003.docx]

**S3 Table. Comparison of the Allplex^TM^ HPV28 and Anyplex^TM^ II HPV28 assays for the specific detection of HR-HPV types in ASCUS samples.**

| **HPV genotypes** | **Population (N=88)** | | | | | |  |
| --- | --- | --- | --- | --- | --- | --- | --- |
|  | **All+**  **n (%)** | **Any+**  **n (%)** | **All+/Any+**  **n** | **All+/Any-**  **n** | **All-/Any+**  **n** | **All-/Any-**  **n** | ***p*** |
| **HPV 16** | 15 (17.0) | 16 (18.2) | 14 | 1 | 2 | 71 | 1.00 |
| **HPV 18** | 5 (5.7) | 4 (4.5) | 3 | 2 | 1 | 82 | 1.00 |
| **HPV 31** | 12 (13.6) | 14 (15.9) | 12 | 0 | 2 | 74 | 0.48 |
| **HPV 33** | 4 (4.5) | 4 (4.5) | 4 | 0 | 0 | 84 | NA |
| **HPV 35** | 3 (3.4) | 3 (3.4) | 3 | 0 | 0 | 85 | NA |
| **HPV 39** | 6 (6.8) | 7 (8.0) | 6 | 0 | 1 | 81 | 1.00 |
| **HPV 45** | 7 (8.0) | 8 (9.1) | 7 | 0 | 1 | 80 | 1.00 |
| **HPV 51** | 13 (14.8) | 12 (13.6) | 12 | 1 | 0 | 75 | 1.00 |
| **HPV 52** | 10 (11.4) | 10 (11.4) | 10 | 0 | 0 | 78 | NA |
| **HPV 56** | 8 (9.1) | 8 (9.1) | 8 | 0 | 0 | 80 | NA |
| **HPV 58** | 5 (5.7) | 7 (8.0) | 4 | 1 | 3 | 80 | 0.62 |
| **HPV 59** | 7 (8.0) | 7 (8.0) | 7 | 0 | 0 | 81 | NA |
| **HPV 68** | 9 (10.2) | 9 (10.2) | 7 | 2 | 2 | 77 | 1.00 |

All+, positive with Allplex^TM^ HPV28; Any+, positive with Anyplex^TM^ II HPV28; All+/Any+, positive with both assays; All+/Any-, Allplex^TM^ HPV28 positive and Anyplex^TM^ II HPV28 negative; All-/Any+, Allplex^TM^ HPV28 negative and Anyplex^TM^ II HPV28 positive; All-/Any-, negative with both assays. *p*, McNemar’s test for paired data. NA = not applicable (if no discordances).
