# Supplementary material for: Comparative evaluation of Allplex HPV28 and Anyplex II HPV28 assays for high-risk HPV genotyping in cervical samples
Source: PLoS One. 2025 Apr 1;20(4):e0320978. doi: 10.1371/journal.pone.0320978 (PMC11960881; doi:10.1371/journal.pone.0320978)
Supplement: S4 Table — All+, positive with AllplexTM HPV28; Any+, positive with AnyplexTM II HPV28; All+/Any+, positive with both assays; All+/Any-, AllplexTM HPV28 positive and AnyplexTM II HPV28 negative; All-/Any+, AllplexTM HPV28 negative and AnyplexTM II HPV28 positive; All-/Any-, negative with both assays. p, McNemar’s test for paired data. NA = not applicable (if no discordances). (DOCX) [file pone.0320978.s004.docx]

**S4 Table. Comparison of the Allplex^TM^ HPV28 and Anyplex^TM^ II HPV28 assays for the specific detection of HR-HPV types in ASCH samples.**

| **HPV genotypes** | **Population (N=9)** | | | | | |  |
| --- | --- | --- | --- | --- | --- | --- | --- |
|  | **All+**  **n (%)** | **Any+**  **n (%)** | **All+/Any+**  **n** | **All+/Any-**  **n** | **All-/Any+**  **n** | **All-/Any-**  **n** | ***p*** |
| **HPV 16** | 2 (22.2) | 1 (11.1) | 1 | 1 | 0 | 7 | 1.00 |
| **HPV 18** | 2 (22.2) | 2 (22.2) | 2 | 0 | 0 | 7 | NA |
| **HPV 31** | 2 (22.2) | 2 (22.2) | 2 | 0 | 0 | 7 | NA |
| **HPV 33** | 1 (11.1) | 1 (11.1) | 1 | 0 | 0 | 8 | NA |
| **HPV 35** | 1 (11.1) | 1 (11.1) | 1 | 0 | 0 | 8 | NA |
| **HPV 39** | 2 (22.2) | 2 (22.2) | 2 | 0 | 0 | 7 | NA |
| **HPV 45** | 0 (0.0) | 0 (0.0) | 0 | 0 | 0 | 9 | NA |
| **HPV 51** | 0 (0.0) | 0 (0.0) | 0 | 0 | 0 | 9 | NA |
| **HPV 52** | 3 (33.3) | 3 (33.3) | 3 | 0 | 0 | 6 | NA |
| **HPV 56** | 1 (11.1) | 2 (22.2) | 1 | 0 | 1 | 7 | 1.00 |
| **HPV 58** | 2 (22.2) | 2 (22.2) | 2 | 0 | 0 | 7 | NA |
| **HPV 59** | 0 (0.0) | 0 (0.0) | 0 | 0 | 0 | 9 | NA |
| **HPV 68** | 1 (11.1) | 2 (22.2) | 1 | 0 | 1 | 7 | 1.00 |

All+, positive with Allplex^TM^ HPV28; Any+, positive with Anyplex^TM^ II HPV28; All+/Any+, positive with both assays; All+/Any-, Allplex^TM^ HPV28 positive and Anyplex^TM^ II HPV28 negative; All-/Any+, Allplex^TM^ HPV28 negative and Anyplex^TM^ II HPV28 positive; All-/Any-, negative with both assays. *p*, McNemar’s test for paired data. NA = not applicable (if no discordances).
